# Relationship between Lighting and Noise Levels and Productivity of the Occupants in Automotive Assembly Industry

**DOI:** 10.1155/2013/527078

**Published:** 2013-10-22

**Authors:** Jafar Akbari, Habibollah Dehghan, Hiva Azmoon, Farhad Forouharmajd

**Affiliations:** Department of Occupational Health Engineering, School of Health, Isfahan University of Medical Sciences, Isfahan 81746-73461, Iran

## Abstract

Work environment affects human productivity and his performance. The aims of this study were to investigate the effects of lighting and noise levels on human productivity in the automotive assembly industry. *Method*. Subjects were 181 workers from different parts of an automobile assembly industry. Illuminance (Lx) at the height of 30 inches from the surface of work station and noise (dBA) were locally measured. Also human productivity by the Goldsmith and Hersey scale (1980) was measured. Data were analyzed by using SPSS v20 Pearson correlation coefficient. *Results*. The results showed that the relationship between noise level and human productivity is negative and significant (*P* < 0.05, *r* = −0.178), but there was no significant relationship between lighting and human productivity (*P* > 0.05). *Conclusion*. Based on the results, in assembly tasks, noise has a negative impact on human productivity, and lighting does not affect this. So, in order to increase employee productivity, noise control and reduction to less than the standard values (less than 85 dB) is necessary.

## 1. Introduction

Human productivity is optimized utilization of human talent, power, and skill to obtain maximum benefits along other factors in organizations and companies [1]. This issue is related to personal, organizational, social, and environmental (noise, lighting, temperature, etc.) factors [2]. In order to increase human productivity, the first step is always creating healthy working environment [3]. Besides having negative effect on human health like desire to increase resting time during working hours, reduction in human concentration, tiredness, and absence from work for being monotonous [4, 5], inappropriate inner environment has also a negative effect on human performance and their comfort during working hours. It has been estimated that inappropriate working environment causes millions of dollars losses [6]. So keeping work environment safe and healthy along with providing human health and comfort and increasing their productivity and performance will increase organization productivity and also will increase quality and quantity of its products and services. 

Physical condition of the workplace in terms of environmental ergonomics, occupational health, and work psychology includes lighting, noise, temperature, relative humidity, and air flow rate [7–9]. Exposure to noise because of work is in connection with negative effect on human health, and its connection with being deaf has been truly approved. In industrial environment, exposure to the noise is in connection with a vast range of physical effects on health, in this regard we can mention heart disease and absence due to work illness and tiredness [10]. Moreover, some studies have shown the connection of response dose of noise exposure and outbreak of psychological distress in industrial workers [11]. So noise has negative effect on job satisfaction of workers [10] and as a result has negative effect on workers' productivity and performance [12]. In addition to direct influences, exposure to the noise in work environment has negative effect on human performance by causing psychological stress [13]. Also the studies have shown that noise exposure in work environment has negative effect on tasks needing concentration [14], performance in investigating and mental tasks needing high mental power [15], and measuring performance in detecting the number of repetitive tasks functions [16].

The direct and indirect influences of lighting intensity on human productivity and capacity as another environmental factor have been examined in various studies, such as improvement of lighting condition which will decrease vision disturbance and neck and shoulder pains [17]. Also improving lighting system causes decrease in eye fatigue and headache [18]. Also negative effect of lighting quality and its efficiency on occupational performance, human temper, health, job satisfaction, and comfort has been studyied. Energetic and high quality lighting provides more adjustment between the person and its work environment [19, 20]. 

Given the above, levels of noise and light by directly and indirectly affecting physical and mental aspects, impact human productivity and performance. So improving its condition will have positive effect on humans and their productivity. Because of assembling vehicles parts with different size and shape, automotive assembly industries require special physical and mental needs and making different aspects of work healthy, like controlling noise and improving lighting system which is an important and effective task. In most of the industries in Iran, noise is considered as a fundamental issue because of not observing controlling issues, which will influence human health and performance. Beside this, task nature in Iran is different from that of other countries, and this difference is because of occupational security, management, and labor law, amount of payment, and social, economic, and cultural conditions which will influence human productivity. So this study aims to investigate whether noise level and lighting intensity can have influence on human productivity regardless of the above-mentioned issues and if it is effective, how much it is, so we can improve the changes of this two parameters for improving human productivity.

## 2. Material and Methods

### 2.1. Subjects

This cross-sectional study has been done on 181 employees from different units of Farman Khodro Sepahan Co. (Isfahan, Iran) in August–November 2012. Different units in this study include administrative, commercial (buying and selling), assembly units of Nissan and Paykan, drilling, production, machining, welding, warehouse, and technoengineering. At first, demographic information including age, sex, height, weight, job experiment, level of education, job, and the related unit of each person has been gathered. Having at least one year of occupational background in related unit was the criterion for entering the test. Other factors include not having heart disease, severe muscular disorder, and diabetes. The factor for discluding the person from the study was his/her reluctance to cooperate and delivery of incomplete questionnaire. 

### 2.2. Noise Level Measurement

For measuring noise level, Noise Level Meter TES-1358 model (TES Electrical Electronic Corp., Taiwan) was located near the stations of the persons to be studied for measuring noise level. Exposure to equivalent noise level (Leq) in each station was 8 hours of daily exposure and 40 hours of weekly exposure. Average level (Leq) was considered for each station. As operator station was fixed, local measurement of noise around the worker was used. Noise level gauges were calibrated by special calibrator, Lutron. Finally, for comparing achieved results by the Standard measurements, standard measurements of Iran noise level (1381, 2nd edition, technical committee of occupational health, Iran management office of work and environment health) were used which are according to the standards of ACGIH. 

### 2.3. Lighting Measurement

To measure lighting in work environment, Light Meter TES-1336 model (TES Electrical Electronic Corp., Taiwan) was used in different units. This device was calibrated in reference laboratory of hygiene center of Isfahan province. Also it was calibrated by zero point calibration method before measuring in units. Measuring total lighting intensity (natural and artificial lighting) by lux was done in workers stations at the height of 30 inches. For comparing the amount of measured lighting intensity to the standard amounts, the standards announced by, 1381, 2nd edition, technical committee of occupational health, Iran management office of work and environment health were used which are according to the Standards of Lighting Engineering Association of North America (IESNA). The amount of lighting defined in this standard for each location has been defined as two amounts, the minimum amount and the recommended amount. The amount of needed lighting by LUX (luman/m^2^) should be according to the recommended amount of lighting.

### 2.4. Human Productivity Assessment

For determining the amount of productivity, 26-questionnaire of Goldsmith and Hersey [21] was used. In compilation of this model, these two researchers tried to determine the issues key factors that can have influence on human performance and also determine these issues in such a way that a manager can use them [21]. According to this, the variable related to effective performance of human will be defined like “ACHIEVE” model. And seven variables including ability, clarity and confidence, help (or support), incentive (or motivation), evaluation, validity, and environment were examined. Scoring this scale will be according to the 5-choice scale of Likert. Validity and stability of this scale were achieved by Kronbakh Alfa coefficient 0.81% by Ashkan Nasirpour et al. (2009) [22].

### 2.5. Statistics

All data were analyzed by using SPSS software (Chicago, IL, USA, version 20.0, SPSS Inc.). Descriptive statistics was used for examining the average and deviation of measured amounts criterion in the statistical group of the studied people. The relation between noise, lighting, and human productivity was examined by Pearson correlation coefficient test. 

## 3. Results

The average of age, experience, height, weight, and BMI (standard deviation) in subjects were 34/11 (1.87) year, 1/7 (0/78) year, 172/6 (2/99) m, 72/17 (5/41) kg, and 24/25 (1/47) kg/m^2^, respectively. The average amount of noise (standard deviation) in a network for all work stations according to dB (standard deviation) was 83/41 (8/46) dB (the range is between 64 and 102 dB). The average amount of lighting in all stations was 310/44, (177/48) (range is between 1310 and 75). Also for the purpose of examining lighting amount in two different seasons, this parameter was measured in August and November 2012, and its average was 320/35 (182/73) and 302/77(173/83), respectively. The average of productivity (standard deviation) was 64/75(15/33) (the range is between 26 and 112). The average of noise, lighting, and human productivity according to the units has been listed in Table 1.

**Figure 1:**
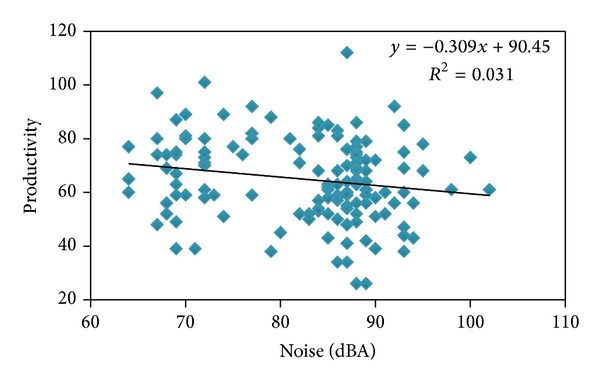
Relationship between noise level (dBA) and human productivity in automotive assembly industry.

**Figure 2:**
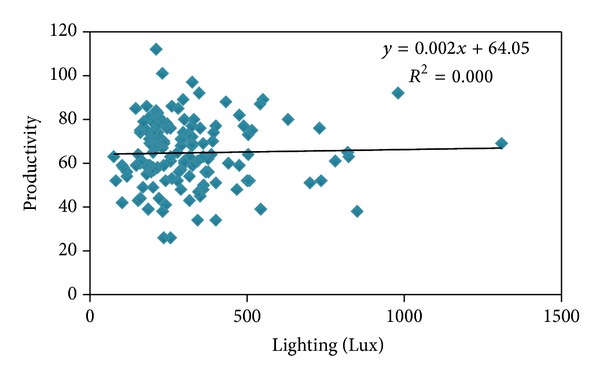
Relationship between lighting (Lux) and human productivity in automotive assembly industry.

The results gained from Pearson correlation coefficient between noise level and productivity level showed that productivity has a significant relationship with noise (*P* = 0.935, *r* = −0.178) (Figure 1). Analysis of data related to lighting and productivity by Pearson correlation coefficient showed that productivity does not have significant relationship with lighting intensity (*P* = 0.753, *r* = 0.027) (Figure 2).

**Figure 3:**
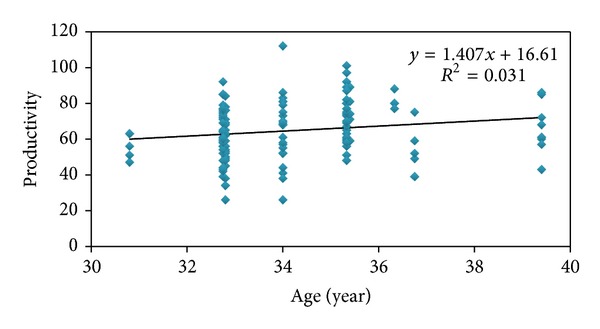
Relationship between age (year) and human productivity in automotive assembly industry.

The result of Pearson correlation coefficient between demographic factors and productivity showed that only age has a significant relationship with human productivity (*P* = 0.035, *r* = 0.177) (Figure 3). Other variables like occupational background, sex, BMI, height, and weight do not have a significant relationship.

## 4. Discussion

Automotive assembly industry by its own nature needs special mental and physical charges. Material handling, sitting at work stations, inappropriate postures and long-time sittings, locating small and big parts, repeated works, and and so forth are risk factors that influence mental and physical health of the human. From the point of view of macroergonomics, one of the most important relations along machine-human relation, system-human relation, and organization-human relation in work environment is the relation of environment and human [23, 24]. Making working environment parameters like noise, lighting, thermal stresses, ergonomics problems, and so on healthy and according to the standards is very important for providing health for workers and increasing their productivity and performance.

In this study, relationship between noise and lighting level with human productivity in automotive assembly industry was examined. The results have shown that in machining, production, Pride assembly, Nissan assembly, drilling, and welding units, the amount of noise level was more than national or international standards (>85 dBA). Also total lighting including natural and artificial lightings was less than the standard amounts in production, warehouse, Nissan assembly, and technoengineering units. The results gained from analysis of Pearson correlation coefficient have shown that there is a significant relationship. This relationship was reverse between noise level and human productivity. The results of this study are aligning with the results of other studies [25–27]. Nowier reports decrease in productivity due to increase of noise (1984-1985). Bergs (2002) reported meaningful difference between noise level and productivity. Also Tafalla and Evans (1997) reported the decrease of productivity due to noise increasing. 

The achieved results of Pearson correlation coefficient did not report any significant relationship between influence of lighting (Lx) and human productivity. Therefore, it has been made clear that lighting amount does not have any influence on human productivity of automobile parts assembly unit and also its changes do not have any influence on productivity of other people in other units. Despite this, most studies have reported improvement of productivity due to improvement of lighting condition. Juslén et al. (2007) have made a research on examining the influence of lighting on the working speed and work quality of electrical devices assembly unit and have reported that production speed in working environment having 1200 lux is 2/9% more than 800 lux and in winter, with higher amount of light, was 3.1% more [28]. Also Niemelä et al. (2002) have reported an increase in productivity due to improvement of lighting condition, aligning with other factors in working environment [29]. According to Hedge (2004), too much luminosity and dazzling in working stations cause a decrease in human performance and productivity. He believes that according to the task the person does and his age, need to lighting will be different [30].

The result of Pearson correlation coefficient has shown that there is a meaningful relationship between personal variables, age, with productivity, and by aging, human productivity will increase. Of course this study was done among the persons of 30/8 to 39/4 years old. Schwab and Heneman have informed that elderly workers are of more productivity than youngers [31]. Skirbekk (2008) has announced that in tasks which need solving a problem, learning, and also speed, aging will decrease productivity while in tasks that experience and verbal ability are important, productivity will slightly decrease or maybe will not at all [32].

## 5. Conclusion

The aim of this study was examining relationship between lighting and noise level on human productivity in automotive assembly industry. Noise level in workplace has negative effect on human productivity which leads to decrease in organization productivity and decrease in quality and quantity of services and products. Therefore, it is recommended to control noise level of workplace and decrease it to standard level (less than 85 dB) to increase comfort and human productivity. Nevertheless, lighting as another environmental factor did not have effect on human productivity, and changes in lighting did not have a relationship with changes of human productivity. 

Among young workers, by aging, the productivity will increase till middle age period. In this case, the skills and experience of working and learning more about the processes and tasks increase human productivity in the workplace. But more study is needed for examining human productivity in different age groups and also determining effective factors of human productivity.

## Figures and Tables

**Table 1:** Average of noise, lighting, and productivity.

Unit	Noise (dBA)	Lighting (Lx)			Productivity
		Actual	Minimum	Proposed	
Tech-en	71.87	195.25	300	500	72.42
Mac	86.00	321.55	300	500	58.07
Ni-as	85.62	275.75	300	500	69.07
War	69.57	164.57	300	500	52.16
Pro	92.50	289.10	300	500	65.88
Pr-as	88.70	305.36	200	300	63.88
Off	70.24	382.55	200	500	72.61
Com	77.57	466.42	300	500	81.25
Dri	89.66	510.66	300	500	56.00
Wel	90.00	203.88	200	300	58.20

Total	83.41	310.44	—	—	64.75

Actual, average measured, minimum, and proposed are standard values; lighting requirements (Lx) must be equal proposed values. Units: Tech-en: technical-engineering, Mac: machining, Ni-as: Nissan assembly, War: warehouse, Pro: production, Pr-as: pride assembly, Off: office, Com: commercial, Dri: drilling, Wel: welding.
